# What are the patients' and health care professionals' understanding and behaviors towards adverse drug reaction reporting and additional monitoring?

**DOI:** 10.1002/pds.5162

**Published:** 2020-11-08

**Authors:** Justina Januskiene, Andrej Segec, Jim Slattery, Georgy Genov, Kelly Plueschke, Xavier Kurz, Peter Arlett

**Affiliations:** ^1^ Pharmacovigilance and Epidemiology Department, European Medicines Agency Amsterdam HS The Netherlands; ^2^ London School of Hygiene and Tropic Medicine London UK

**Keywords:** additional monitoring, adverse drug reactions, black triangle, impact, pharmacoepidemiology, pharmacovigilance

## Abstract

**Background:**

The additional monitoring (AM)/black triangle concept is aimed to enhance ADR reporting for certain types of medicinal products for which the safety profile is less well established.

**Purpose:**

The objective of this survey was to assess (a) attitudes towards ADR reporting and reasons for not reporting an ADR and (b) awareness of AM among HCPs, patients or their careers in EU countries.

**Methods:**

An online questionnaire which was available in all EU languages was completed by 2918 responders coming from all EEA countries.

**Results:**

The main factors motivating to report an ADR were severity or novelty of the reaction or novelty of the medicine. The main factors for not reporting an ADR was the fact that the ADR is already known (35%), the ADR was not serious (18%) or reporter was not sure if the ADR was related to the medicine (15%). Half of the respondents indicated that they have seen AM statement before. Thirty percent of the responders had correct understanding of the AM concept while 20 % misunderstood the concept.

**Conclusion:**

Underreporting occurs but it seems this is because of reporter's prioritisation towards certain type of ADRs. AM aims to increase reporting for certain medicines, however, approximately half of responders have seen the AM symbol before and 20% of all responders (independent of their previous awareness) misunderstood the concept.

**KEY POINTS**

- Pan European survey investigated attitudes and awareness of adverse drug reaction (ADR) reporting and additional monitoring (AM).
- Reporters were most likely to reports ADRs that were fatal, serious, novel or were associated with a new medicine, and not report the ADRs that are already known, not serious, or if reporter was not sure if the ADR was related to the medicine.
- Awareness of AM varies between different respondent groups, the greatest being among pharmacists, and one in five respondents misunderstood the concept of AM.
- Continued awareness campaigns on ADR reporting and AM, especially targeting health care professionals, are needed.

## 1 INTRODUCTION

**FIGURE 1:**
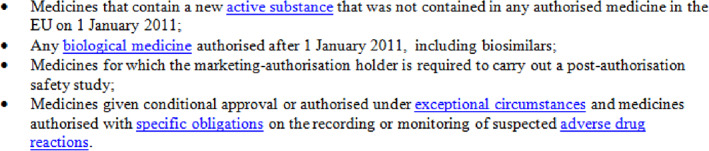
Mandatory scope of additional monitoring^3^, ^4^ [Colour figure can be viewed at wileyonlinelibrary.com]

**FIGURE 2:**
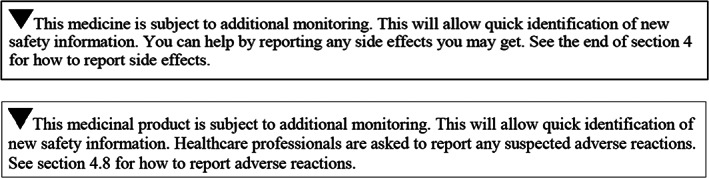
Black triangle symbol and statement included in the summary of product characteristic (top) and package leaflet (bottom) of products under additional monitoring^3^, ^4^

The EU system of pharmacovigilance was significantly strengthened in 2012 with the introduction of new legislation delivering new processes, responsibilities and tools.^1^ Adverse drug reaction (ADR) reporting is an essential part of the surveillance after introducing new medicines to the market. Underreporting is well recognised and is reported to be as high as 94%.^2^ Increasing ADR reporting aims to improve detection of new safety issues. Various legislative changes have been introduced to increase ADR reporting.^1^, ^3^, ^4^ One of these changes was the additional monitoring (AM) system, which was introduced by the 2010 pharmacovigilance legislation to enhance ADR reporting for certain types of medicinal products for which the safety profile is less well established and by so doing, to increase detection of safety issues.^3^, ^4^ Figure 1 shows which medicinal products fall under the mandatory scope of AM. Since April 2013 every medicine subject to AM must display an inverted black triangle and accompanying statement in product information, as shown in Figure 2.

Since 2013 various activities have been undertaken by national competent authorities (NCA) and by the European Medicines Agency (EMA) to increase the awareness of additional monitoring. NCAs' web‐sites, publications and Health Care Professional (HCP) conferences were the most frequent activities.^5^

Our study aimed to better understand patients' and healthcare professionals' awareness of ADRs reporting in general and in particular their understanding of the AM concept and reporting of ADRs for medicinal products under AM. While there are many studies investigating the patterns in ADR reporting (or underreporting) overall, much less is known about the impact of AM.^2^, ^6^, ^7^ Therefore our survey was among the first investigations into HCPs' and patients' understanding of the AM concept.^6^, ^8^ This research was mandated by the Pharmacovigilance Risk Assessment Committee (PRAC) impact strategy group^9^ and was conducted to support the EMA and NCA contribution for the European Commission (EC) report on the experience with the list of products subject to additional monitoring^1^.^5^

## 2 DATA AND METHODS

In September 2017, EMA conducted an online survey of patients' and HCPs understanding of ADR reporting and the AM concept. Prior to its release it was user‐tested internally by 34 EMA staff members (half were without medical training). The survey was then translated into all official European Union (EU) languages and published on the EMA's website (hosted on the EU survey tool https://ec.europa.eu/eusurvey/home/about). The Patients and Consumers Working Party and HCP Working Group of the Committee of Medicinal Products for Human Use (CHMP) and EMA helped to design and disseminate this survey through their networks. An electronic link to the survey was shared via emailing lists, patients' organisations social media platforms and their websites. The survey was aimed at HCPs, patients or their carers but was also accessible to the general population. It was open for responses for five weeks from September 4, 2017 until October 9, 2017.

The survey (section 1 in the supplementary material) had three sections. A socio‐demographic part where data about country, gender, age and type of responder were collected. Responders were asked to choose the role in which they prefer to respond. Responder categories included nurse, physician, pharmacist or other HCPs (categories classified as HCPs in the results) or patient/carer or member of the public (categories classified as non‐HCPs in the results). HCPs were specifically asked where they were practicing (with an aim to establish whether they see patients and prescribe medicines on a regular basis) and non‐HCPs were specifically asked how many different medicines they had taken last month. The second section included questions on actual ADR reporting and attitudes to ADR reporting. The third section was related to AM concepts starting with awareness of AM, understanding of AM and actual reporting behaviour for AM products, and specifically whether reporting was affected by the black triangle/AM status.

The survey consisted of eight multiple choice questions and two open‐ended questions to identify reasons for not reporting ADRs and measuring of AM understanding (this allowed up to 200 characters).

### 2.1 Analysis of responses

Responses to open‐ended question on AM understanding (“In your opinion, what does the black triangle and the accompanying statement mean?”) were translated to English (if needed) and assessed by two independent evaluators. In case of disagreement between the two evaluators, the open question response was sent to the third evaluator. The incidence of such disagreement was very low (<1%). All responses were classified into five categories of understanding, see Table 1 below. These five categories were created by analysing the sample of 100 responses.

**TABLE 1:** Classification of the responses to the open field question on AM understanding

Classification	Criteria
Correct understanding	Answers mentioning ARs reporting, novelty of the drug
Insufficient information	Dots, commas, symbols, untranslatable abbreviations and also simply the phrase “additional monitoring”
Misunderstanding	Elements that are clearly not a definition of the black triangle, for example, “a more toxic drug”, “drug with no clinical trial data” or other
No understanding	“I do not know” or similar
Not responded	Field left blank

The structured responses were described using counts and frequencies and compared using non‐parametric tests for independent grouped categorical data (chi‐square tests and P‐value). MS Excel software was used.

## 3 RESULTS

In total 2918 responses were received, 2862 of them being from EEA countries (median: 40 per country, range: 4‐569) and 56 from non‐EEA countries. The largest number of responses was received from Portugal, Germany and Italy likely due to more active promotional activities by patients' organisations in these countries. Overall, 1385 (47%) respondents identified themselves as non‐HCPs (1138 (39%) as patients or carers and 247 (8%) as members of the public), and 1533 (53%) respondents were HCPs (365(13%) physicians, 848 (29%) pharmacists, 212 (7%) other HCPs and 108 (4%) nurses). The median age of non‐HCPs was 45 years (range 16‐81) and for HCPs 40 years (range 18‐82). Sixty six percentage of all responders to the survey identified themselves as females, 30% as males and 4% did not respond to this question.

**FIGURE 3:**
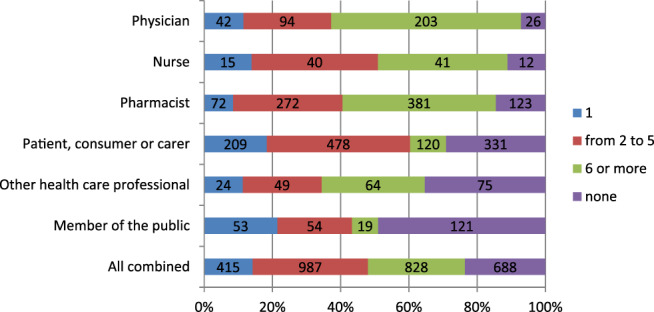
Number of ADRs observed/experienced by various types of responders (n = 2918) [Colour figure can be viewed at wileyonlinelibrary.com]

Most of the responders (76%, n = 2230) indicated that they have observed in their patients (85%, N = 1297 for HCPs) or experienced themselves (67%, n = 933 for non‐HCPs) at least 1 ADR during their lifetime. Responses varied greatly depending on responder's qualification (only 7% of physicians never observed an ADR, while in members of the public and patient groups this proportion was 49% and 29%, respectively) (Figure 3).

**FIGURE 4:**
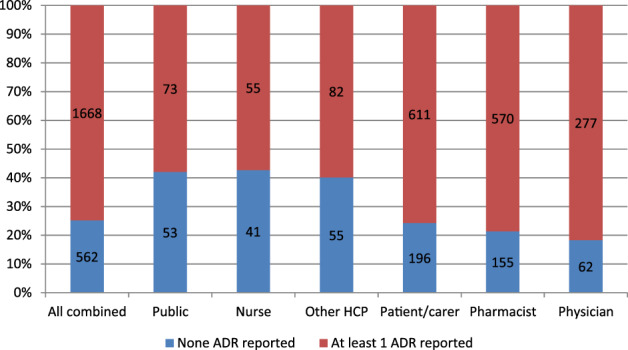
Numbers of (%) responders who reported an ADR at least once, when the number of observed/experienced ADRs was 1 or more (n = 2230) [Colour figure can be viewed at wileyonlinelibrary.com]

The 2230 responders that had observed or experienced an ADR, were asked, how many times they reported an ADR. 76% of HCPs and 73% non‐HCPs indicated that they reported at least once (n = 1668 in total). Figure 4 shows the proportion of responders who reported an ADR at least once, when the number of observed/experienced ADRs was 1 or more. This suggests underreporting of ADRs varied by responder type and ranged between at least 19% and 42%.

Among ADR reporters (n = 1668), 14% (n = 227) reported an ADR for a product identified with a black triangle (this question was populated only if the responder did not select “none” in the question on how many ADRs they had ever reported). Those who responded positively to this question (n = 227) were asked if the black triangle influenced their decision to report the ADR. Thirty seven percentage of this group indicated that the black triangle was an influencing factor.

**FIGURE 5:**
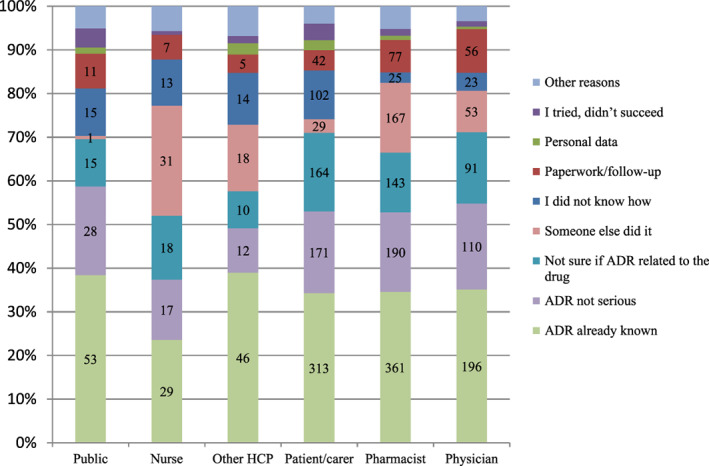
Reasons for not reporting an ADR (n = 3532, multiple choice question) [Colour figure can be viewed at wileyonlinelibrary.com]

The main reasons to for not reporting an ADR were investigated. The results are shown in Figure 5 below. Our results indicate that the main reasons for lack of ADR reporting is the fact that the ADR is already known (35%), the ADR is not serious (18%) or the reporter is not sure if the ADR was related to the medicine (15%). Six Hundred and thirty eight responders indicated that they reported all reactions that they have observed/experienced.

The question on reasons for not reporting an ADR was a multiple choice one with an open field to be filled in if the responder selects “other”. A total of 95 HCPs and 74 non‐HCPs specified other reasons for not reporting. Among HCPs, time constraints (n = 30), technical problems (n = 16) were most common responses, while among non‐HCPs the most frequent response (n = 44) was unsupportive physician (physician who did not believe the patient when told about an ADR or refused to report it). Six responses stated that they did not know that they can report ADRs and six reported that they expected HCPs to report their ADRs.

**FIGURE 6:**
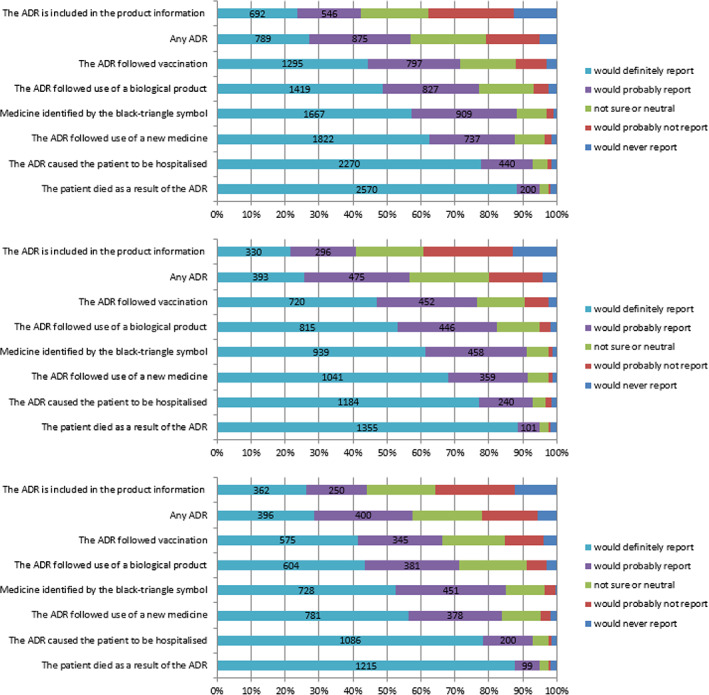
Attitudes towards reporting various types of ADRs, all respondents combined (top), HCPs (middle), and non‐HCPs (bottom) [Colour figure can be viewed at wileyonlinelibrary.com]

The respondents' attitudes were then investigated with the question “How likely are you to report the following types of reactions” with a matrix table listing various types of ADRs. As presented in Figure 6, the main factors motivating to report an ADR were severity or novelty of the reaction or novelty of the medicine. Fifty seven percentage of HCPs would probably or definitely report any ADR; this percentage was over 70% for vaccines and biological products.

A total of 88% indicated that they would definitely or probably report an ADR for a medicine identified with a black triangle which mirrors the answers to the question on new medicines (also 88%). Serious reactions (leading to hospitalisation or fatal outcomes) were overall the most likely to be reported (93% and 95%, respectively).

Reporting attitudes were different among different respondent types (*P* < .001). Physicians and pharmacists are less likely than patients and members of the public to report “any ADR” but more likely to report fatal and serious ADRs (over 97% and 93%, respectively) and for new medicines and those with a black triangle (93% and 90%, respectively).

With regards to the AM symbol (black triangle), 51% of the 2918 responders indicated that they had seen the black triangle and the accompanying statement before. Awareness varied between different respondents, the lowest was among patients (30% reported that they had seen it before) and the highest among pharmacists (83%).

**FIGURE 7:**
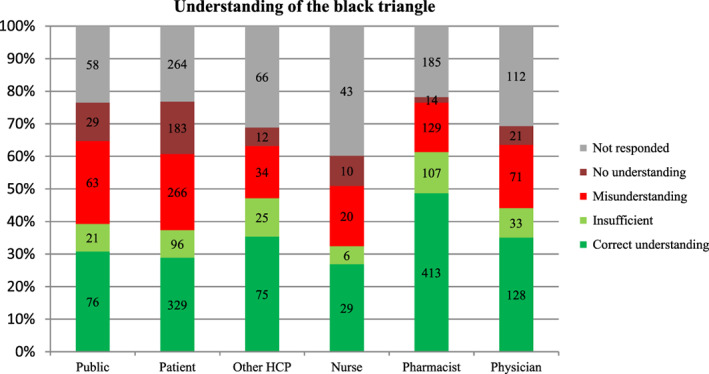
Understanding of black triangle and AM concept by different responders (n = 2918) [Colour figure can be viewed at wileyonlinelibrary.com]

Regarding the understanding of the message accompanying the black triangle, in total, 36% (1050 of 2918 responders) of responses showed an understanding classified as correct, while 20% (583) of the responses were classified as misunderstanding, 9% of replies showed no understanding, 10% of responses had insufficient information for proper classification, and 25% did not responded to this question (see Figure 7 for more details). After excluding 56 responses from non‐EU countries, the results remained unchanged. Twenty percent of non‐EU responders misunderstood the AM concept and 36% had a correct understanding.

As shown in Figure 7, the level of understanding was different among different types of responders, being greatest among pharmacists (45%), physicians (35%), and other HCPs (35%) and lowest among patients (29%), general public (30%) and nurses (27%). The level of understanding also varied greatly depending on previous awareness of the black triangle: 48% of all responders who had seen black triangle before, had correct understanding, compared to 24% of those who have not seen it before.

Among responses classified as “misunderstanding”, the most prevalent themes were perceived safety concerns (eg, “drug is toxic”, “drug causes more side effects than other drugs”), lack of safety data (eg, “trial drug”, “drug marketed without clinical trial”, “unknown safety profile”) and a combination of other themes (“narrow therapeutic index drug”, “a need of patient monitoring”, “keep out of reach of children”, “careful driving”), or other (“washing instruction”, “drop down list”). The themes in the responses were also similar between HCPs and non‐HCPs.

## 4 DISCUSSION

To our knowledge, this study is the first to measure the understanding of the concept of additional monitoring and its impact on ADR reporting across Europe. One of the main limitations of this study is selection bias. Due to the nature of dissemination of the survey the responders are likely to be more aware of medicines and AM than the general population. Because of the technical limitations of the EU survey tool we are not able to calculate the response rate to the survey. Figures showing the proportion of ADR reported should be interpreted in this context. Responses to the questions on attitudes towards ADR reporting (how likely you would report various ADRs) may also be subject to response bias,^10^ with responders presenting a more favourable responses, likely inflating the proportion of responders who responded that they would report various ADRs/report all ADRs Also, responders were asked to choose in which role they prefer to respond (HCP or non‐HCP) however, we have no data on whether HCPs chose to respond to the survey as non‐HCP or vice versa. Another limitation is that three countries (Portugal, Germany, and Italy) accounted for the 49% of the responses. Awareness of AM in these countries was better than the survey average, with 43% of responders from Germany and 33% in both Portugal and Italy having a good understanding of AM. In the UK, the black triangle concept existed before the AM was introduced in the EU. Awareness of AM in the UK was not better than the average for our survey (37% had correct understanding of AM and 23% misunderstood the concept). However, it is acknowledged that the criteria for AM are different from the ones that were used in the UK black triangle scheme.

The main reasons for not reporting an ADR were based on a lack of certainty, namely not being sure if an ADR is previously known or if it is related to the medicine. Our results show that HCPs and non‐HCPs are likely to report an ADR if the reaction is serious and not previously known and these findings are in line with previous research.^6^, ^7^, ^8^ Only 8% of non‐HCPs indicated that they have not reported an ADR because they did not know how to do so (see Figure 5). This is an interesting finding which is not in line with previous research, presumably because of the selection bias of the respondents. The systematic review done by Rania Al Dweik^11^ highlighted that poor patient awareness of available reporting systems was the main barrier for reporting of ADRs. According to that study the main motive for patient reporting of ADRs was to prevent others from experiencing the same ADR. This indirectly mimics our finding that previously unknown ADRs are more likely to be reported.

Not every medicinal product is subject to AM and is identified with the black triangle and the symbol was only introduced relatively recently. Therefore it is reasonable that many patients or members of the public responding to the survey have never used (or seen) a medicine with a black triangle. In our study, we found that 30% of non‐HCPs are aware of AM/black triangle, higher than in previous research. In the survey conducted by EURORDIS^8^ on the new pharmacovigilance system 20% of respondents (patients) indicated that they had seen a black triangle. However, this question was asked differently in the EURORDIS survey, responders needed to select one of their medicines and respond to the questions based on the leaflet information of this medicine. Our survey asked a more general question “have you ever noticed a black‐triangle symbol?” A survey conducted by O'Callaghan et al. in Ireland on HCPs on biological medicines also contained questions on AM. Similarly to our survey, the best awareness of AM was among pharmacists.^6^

Perception and understanding of AM was significantly different between those who saw the AM symbol before participating in our survey and those who did not (48% vs 24%). This demonstrates that awareness campaigns are likely beneficial to raise knowledge about this scheme and to avoid misinterpretation.

On another hand, if many patients are unlikely to ever encounter an AM medicines, expending a large amount of resources to raise awareness among patients/non‐patients (healthy population) may not yield the best results. A good understanding of AM concept among HCPs is important. HCPs are in direct contact with patients and can explain to them about AM also they are more likely report ADR for AM medicines, as most AM medicines are prescription medicines and usually novel therapies. This suggests a strong role for prescribers and pharmacists in educating patients about ADRs and their reporting. Therefore targeted awareness campaigns to HCPs might improve reporting practice, especially for AM products. This in turn allows the reporting of suspected reactions, detection and assessment of potential risks related to the use of medicines and thus optimising the benefit‐risk balance for patients.

It is difficult to conclude whether AM/black triangle had an effect on actual ADR reporting behaviour, as a very limited number of responders replied that they reported an ADR for an AM medicine. Another study conducted in the United Kingdom on the impact of the black triangle label on prescribing of new drugs concluded that AM was unlikely to change prescribing practice.^12^

The survey was available in all EU countries, and to our knowledge, this is the largest one on the topic to date. A wider‐reaching survey, not only online, but also paper‐based for those unable to use digital media and reaching a wider range of patients might give more representative estimates on AM awareness by reflecting a less specialised profile of responder.

## CONFLICTS OF INTEREST

The views expressed in this article are the personal views of the author(s) and may not be understood or quoted as being made on behalf of or reflecting the position of the EMA or one of its committees or working parties.

## Supporting information


**Appendix** **S1**. Supporting Information.

## Endnote

^1^
Article 23(4a) of Regulation (EC) No 726/2004 states that the EC shall present by fifth June 2018 to the European Parliament and the Council a report on the use of the list of products subject to AM list. If considered appropriate, the Commission shall, on the basis of that report, and after consultation with the Member States (MSs) and other appropriate stakeholders, present a proposal in order to adjust the provisions relating to the AM list referred to in REG 23.

## References

[1] Santoro A , Genov G , Spooner A , Raine J , Arlett P . Promoting and protecting public health: how the European Union pharmacovigilance system works. Drug Saf. 2017;40(10):855‐869. 28735357 10.1007/s40264-017-0572-8 PMC5606958

[2] Hazell L , Shakir SAW . Under‐reporting of adverse drug reactions: a systematic review. Drug Saf. 2006;29(5):385‐396. 16689555 10.2165/00002018-200629050-00003

[3] Guideline on good pharmacovigilance practices Module X—Additional monitoring. Retrieved from. http://www.ema.europa.eu/docs/en_GB/document_library/Scientific_guideline/2013/04/WC500142282.pdf

[4] Regulation (EC) No 726/2004 of the European Parliament and of the council of 31 March laying down Community procedures for the authorisation and supervision of medicinal products for human and veterinary use and establishing a European Medicines Agency, Official Journal L–136, p. 1–33. Retrieved from http://eur-lex.europa.eu/LexUriServ/LexUriServ.do?uri=OJ:L:2004:136:0001:0033:en:PDF

[5] Report from the Commission to the European Parliament and the Council on the national and European Medicines Agency experience regarding the list of medicines for human use subject to additional monitoring. Retrieved from https://ec.europa.eu/transparency/regdoc/rep/1/2019/EN/COM-2019-591-F1-EN-MAIN-PART-1.PDF

[6] O'Callaghan J , Griffin BT , Morris JM , Bermingham M . Knowledge of adverse drug reaction reporting and the pharmacovigilance of biological medicines: a survey of healthcare professionals in Ireland. BioDrugs. 2018;32(3):267‐280. https://www.ncbi.nlm.nih.gov/pubmed/29721705. 29721705 10.1007/s40259-018-0281-6 PMC5990561

[7] Avery AJ et al. Evaluation of patient reporting of adverse drug reactions to the UK'Yellow card Scheme': literature review, descriptive and qualitative analyses, and questionnaire surveys. Health Technol Assess. 2011;15(20):1‐234. 10.3310/hta15200. 21545758

[8] Presentation by Francois Houÿez “What does the new PhV system mean for patients in real life?” Retrieved from https://www.eurordis.org/sites/default/files/Eurordis_patients_and_pharmacovigilance.pdf

[9] PRAC strategy on measuring the impact of pharmacovigilance activities (Rev 1). Retrieved from http://www.ema.europa.eu/docs/en_GB/document_library/Other/2016/01/WC500199756.pdf

[10] Thea F , Mortel Van de . Faking it: social desirability response bias in self‐report research. Aust J Adv Nursing. 2008;25(4):40‐48.

[11] Al Dweik R et al. Factors affecting patient reporting of adverse drug reactions: a systematic review. Br J Clin Pharmacol. 2017;83(4):875‐883. 27868226 10.1111/bcp.13159 PMC5346870

[12] Horton DB et al. Impact of the black triangle label on prescribing of new drugs in the United Kingdom: lessons for the United States at a time of deregulation. Pharmacoepidemiol Drug Saf. 2017 Nov;26(11):1307‐1313. 10.1002/pds.4304. 28857309 PMC5670006

