# Supplementary material for: What are the patients' and health care professionals' understanding and behaviors towards adverse drug reaction reporting and additional monitoring?
Source: Pharmacoepidemiol Drug Saf. 2020 Nov 8;30(3):334–41. doi: 10.1002/pds.5162 (PMC7894330; doi:10.1002/pds.5162)
Supplement: Supplementary file 1 — Appendix S1. Supporting Information. [file PDS-30-334-s001.docx]

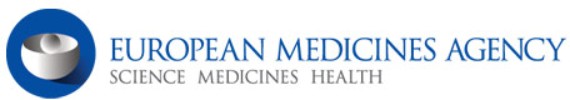


Survey on safety of medicines and reporting of adverse drug reactions

Fields marked with * are mandatory.

Thank you for participating in this survey, which will take approximately 5 to 10 minutes of your time to complete. Your answers will help us understand the awareness of patients/consumers and healthcare professionals regarding the need and the way they can report adverse drug reactions (side effects). The results will be analysed by the European Medicines Agency and a report containing summary information will be provided to the European Commission (DG SANTE) and will be further disseminated publicly.

Please answer the questions to reflect your current practice and experiences and do not submit multiple submissions.

The questionnaire does not require you to disclose your identity. We will collect and analyse your responses but we will not link them to any other personal information you provide. Collected data will be kept one year and then destroyed. For security reasons and to manage the orderly participation in the survey, the survey platform will record IP addresses for every server request accessing the questionnaire. If you have any questions about this survey, please contact [survey@ema.europa.eu](mailto:survey@ema.europa.eu)

# About you

-
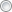
1. Where do you currently live? Austria


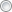

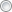

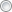

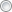

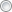
Belgium Bulgaria Croatia Cyprus


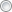

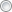

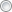
Czech Republic Denmark Estonia


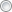

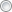

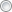

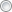

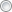

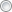

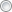

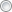

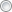
Finland France Germany Greece Hungary Iceland Ireland Italy Latvia

Liechtenstein


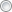

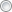

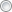

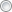

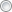

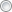

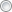

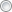

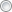
Lithuania Luxembourg Malta Netherlands Norway Poland Portugal Romania


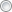

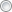
Slovak Republic Slovenia


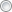

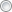
Spain Sweden


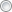
United Kingdom Other country

Other - specify

- 1.
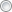

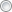
What is your gender? Female

Male


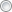
Other or prefer not to answer

- 1. What is your age?

*Only values between 16 and 110 are allowed*

years

-
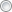
4. Are you … (if more than one apply, please choose one role in which you prefer to answer the survey). Patient, consumer or carer


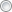

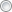

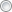

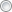
Member of the public Physician Pharmacist

Nurse


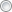
Other health care professional

-
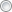

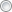

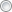
4.1. Which sector do your work in? Community or primary care Hospital or secondary/tertiary care Academia


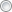

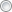

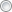
Pharmaceutical industry Regulatory authority/government Other – specify

Other - specify

*100 character(s) maximum*

- 4.1. In the last month, how many different medicines have you taken? (please count prescribed medicines and OTC medicines, but do not count food supplements and vitamins).

*If you are a carer, please reply on behalf of the person you care for.*

*Only values between 0 and 100 are allowed*

# The next questions relate to adverse drug reactions caused by medicines, also known as side effects. Sometimes patients experience adverse drug reactions and they report them to their health care providers or to a medicines authority or a pharmaceutical company. Healthcare professionals also identify adverse drug reactions themselves.

-
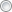
5. How many adverse drug reactions, if any, have you ever observed in patients? 1


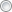
2-5


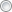

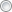
6 or more None

-
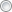
5. How many adverse drug reactions (side effects) have you or the person you care for ever experienced? 1


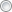
2-5


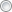

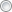
6 or more None

- 5.1. How many times have you reported an adverse drug reaction (side effect) to an authority or a pharmaceutical company?


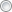

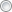

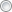
Once 2-5


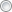
6 or more Never

- 5.1. How many times have you reported an adverse drug reaction (side effect) to your doctor/pharmacist

/nurse, to a medicines authority or a pharmaceutical company?

Once 2-5

6 or more Never

- 5.2. In case you have not reported all adverse drug reactions you observed/ experienced, what were the main reasons to for this? Please select all that apply.


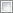
 Not applicable (I reported all reactions that I have observed/experienced)
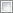
 Someone else reported it (e.g. other doctor)


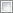
 I did not want to deal with paperwork/follow-up questions
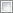
 I did not want to share my personal data


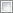
 I was not sure if the adverse reaction was related to the drug


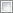
 The adverse drug reaction was already known (included in the product information)
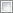
 The adverse drug reaction was not serious


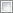
 I did not know how to report it


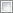
 I tried to report, but didn’t succeed (please give a reason why in the open field section below)
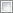
 Other reasons

Other reasons (open field, up to 250 characters)

*250 character(s) maximum*

1. How likely are you to report the following types of reactions on a scale of 1 to 5?

|  | 1  (would never report) | 2 (would probably not report) | 3 (not sure or neutral) | 4 (would probably report) | 5 (would definitely report) |
| --- | --- | --- | --- | --- | --- |
| ***** Any adverse drug reaction | 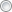 | 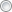 | 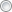 | 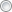 | 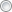 |
| ***** The patient died as a result of the reaction | 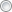 | 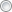 | 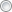 | 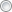 | 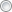 |
| ***** The reaction caused the patient to be hospitalised or needing significant medical treatment | 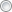 | 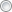 | 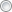 | 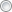 | 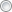 |
| ***** The reaction is included in the product information as a known adverse reaction for the drug | 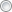 | 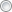 | 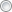 | 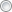 | 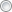 |
| ***** The reaction followed vaccination | 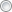 | 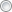 | 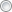 | 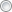 | 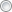 |
| ***** The reaction followed use of a biological product (e.g. insulin, blood clotting factor, etc.) | 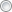 | 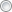 | 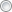 | 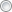 | 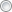 |
| ***** The reaction followed use of a new medicine |  |  |  |  |  |

# Additional monitoring

1. Have you ever noticed a black-triangle symbol and the accompanying statement shown below in information about medicines?

## This medicinal product is subject to additional monitoring. This will allow quick identification of new safety information. Healthcare professionals are asked to report any suspected adverse reactions. See section 4.8 for how to report adverse reactions.

**This medicine is subject to additional monitoring. This will allow quick identification of new safety information. You can help by reporting any side effects you may get. See the end of section 4 for how to report side effects.**

- Please tick all that apply.

Yes, in the summary of product characteristics/in the package leaflet

Yes, in educational or promotional material (e.g. booklets, patient education cards) Yes, in publications such as formularies or bulletins

Yes, in other information sources (e.g. drug information web-sites) No

- 7.1. Did you understand the meaning of the black-triangle symbol and its explanation? Yes

Not sure No

1. In your opinion, what does the black triangle and the accompanying statement mean?

*250 character(s) maximum*

# Additional monitoring

## This medicinal product is subject to additional monitoring. This will allow quick identification of new safety information. Healthcare professionals are asked to report any suspected adverse reactions. See section 4.8 for how to report adverse reactions.

**This medicine is subject to additional monitoring. This will allow quick identification of new safety information. You can help by reporting any side effects you may get. See the end of section 4 for how to report side effects.**

- 9. Based on the information provided in the statement above, how likely are you to report an adverse drug reaction to a medicine identified by the black-triangle symbol?

Would never report

Would probably not report Not sure or neutral

Would probably report Would definitely report

- 10. Have you ever reported an adverse drug reaction for a product identified by the black-triangle symbol? Yes, more than once

Yes, once No

- 10.1. Did the black triangle influence your decision to report this adverse reaction? Yes

No

These are all the questions we had for you today. Many thanks for your participation.

Further information on additional monitoring and reporting of adverse drug reactions can be found here: http://www.ema.europa.eu/ema/index.jsp?curl=pages/special_topics/document_listing

/document_listing_000365.jsp&mid=WC0b01ac058067bfff
